# Hidden diversity: Phylogeography of genus *Ototyphlonemertes* Diesing, 1863 (Ototyphlonemertidae: Hoplonemertea) reveals cryptic species and high diversity in Chilean populations

**DOI:** 10.1371/journal.pone.0195833

**Published:** 2018-04-26

**Authors:** Cecili B. Mendes, Jon L. Norenburg, Vera N. Solferini, Sónia C. S. Andrade

**Affiliations:** 1 Laboratório de Diversidade Genômica, Departamento de Genética e Biologia Evolutiva, IB (USP), São Paulo, SP, Brazil; 2 National Museum of Natural History, Smithsonian Institution, Washington, DC, United States of America; 3 Laboratório de Diversidade Genética, Departamento de Genética, Evolução Microbiologia e Imunologia, IB (UNICAMP), Campinas, SP, Brazil; National Cheng Kung University, TAIWAN

## Abstract

*Ototyphlonemertes* is a cosmopolitan genus of meiofaunal nemerteans. Their morphological characters are insufficient to reliably identify and delimit species. Consequently, some of the species are considered cosmopolitan despite anticipated low dispersion capability of the adults and a short planktonic larval phase. Indeed, recent studies show that some species actually comprise cryptic species, and populations are connected by stochastic events of long-distance dispersion. Based solely on morphological traits, a Lactea and a Pallida morph of *Ototyphlonemertes* are recognized here from collections at eight and five locations respectively along the Chilean coast. To assess the phylogeographic patterns of their populations, two mitochondrial markers (COI and COX3) of 162 specimens of Lactea and 25 of Pallida were sequenced. Final sequences are 605bp and 362bp for COI and COX3, respectively. Results from phylogenetic and haplotype network analyses suggest that the Lactea morph comprises up to three independent evolutionary units (one with only COX3 sequences). A COI gene tree including other previously published *Ototyphlonemertes* sequences groups the Chilean Lactea with other Lactea, while the Chilean Pallida is grouped with other Pallida. Different structuring and gene flow patterns found for the four groups support the hypothesis that these are four independent evolutionary entities with different ecological, dispersal and demographical characteristics.

## Introduction

### Meiofauna dispersal

The meiofauna comprises a huge variety of animals representing most of the invertebrate phyla. These animals can pass through a 500-μm mesh and, due to their taxonomic and ecological complexity, represent a very important component of marine ecosystems [1, 2, 3].

Unlike sessile organisms, which can actively disperse only during the larval stages, meiofaunal animals usually lack larval stages or larvae stay in the plankton for short periods [4], but adult dispersion can play a major role in connecting populations. For instance, harpacticoid copepods actually swim into the water column when the water velocity is slower [5, 6, 7]. Other animals, however, have body structures, such as the adhesive glands found in many marine meiofaunal worms, for attaching to sediment particles to avoid transport [8, 9, 10].

Most meiofaunal animals are inferred to have low dispersal ability; however, many of these species are widely distributed, and some are even considered cosmopolitan [11, 12, 13, 14]. Recent studies with these taxa show most of them to comprise morphologically cryptic species, probably isolated by the distance between populations (e.g., [15, 16, 17, 18, 14, 19, 20]), but some of those still have ranges of more than 5,000 km, likely due to adult transport with or without sediment grains in stochastic events [14, 20].

### The genus *Ototyphlonemertes*

*Ototyphlonemertes* Diesing, 1863 is a cosmopolitan genus of interstitial nemerteans. They are unique among nemerteans (with the exception of two unconfirmed reports) in possessing a pair of cerebral statocysts. They are stenoecius and most commonly live in littoral or shallow sublittoral moderately sorted coarse sand and/or shell-hash. The adults avoid being swept away by waves by actively migrating deeper into the sediment [21, 22] and, in some species, using adhesive glands to attach to the sand grains [23].

*Ototyphlonemertes*, like many nemerteans, have a body with a simple morphology and no appendages, making the taxonomic delimitation of species difficult. Most of the species in the genus are identified based on some or all of the following morphology: characteristics of the statocysts; presence/absence of cerebral organs; sensory bristles and adhesive structures; as well as, especially, localization and size of the proboscis and characteristics of its components such as the stylet, accessory stylet pouches, papillae, and proboscis regions. Though there is the overall appearance of miniaturization and some simplification, as seen in other interstitial meiofauna, in the aforementioned features *Ototyphlonemertes* has significantly greater morphological disparity across the genus than other speciose nemertean genera (JLN pers obs). Unfortunately, some of those characteristics can be variable within even a genetically delimited species [20]. In addition, many species, especially those named early in history, lack unambiguous morphological diagnoses. Several of those species are recorded worldwide. To facilitate identification, Envall and Norenburg [23], while recognizing the potential for non-monophyly, established six morphotypes with unique characteristics: Pallida, Fila, Lactea, Cirrula, Duplex and Macintoshi. For the most part these are unambiguous morphotypes but, unsurprisingly, two of these have been found to be paraphyletic or polyphyletic in recent genetic study [20].

Recent studies assessing the genetic structure of these species showed that many have a smaller distribution than was inferred by morphological studies [20, 23, 24]. Some species, notwithstanding, can have long-distance dispersal, as, for instance, *Ototyphlonemertes parmula* Corrêa, 1950 [18]. Three studies covering five *Ototyphlonemertes* morphotypes found some lineages geographically restricted, while others showed evidence of long-distance gene flow [17, 18, 20]. A lineage of the Parmula morph present in both the Caribbean and Atlantic coasts of Florida, showed long-distance dispersal of as much as 2,000 km as well as sympatry of cryptic putative sister lineages, which could have been caused via dispersal of adults by storms and/or sediment transport [18]. Some of the morphotypes along the Brazilian coast appear to be species complexes, of which some showed evidence of long-distance dispersal [17]. The presence of sympatric cryptic species indicates that the major isolation force might be environmental change causing local extinction, rather than the geographic distance between populations [20].

### Chilean coastal waters

The Chilean coast is more than 4,000 km long and has a complex system of ocean currents. The marine populations present along this coast have different patterns of connection and dispersion. Many coastal species seem to have gene flow restricted by a biogeographical transition zone around 30° S latitude, dividing the coast into North and South (e.g., [25, 26,27, 28]). This barrier is manifested as changes in wind patterns, upwelling regimes and associated variability in sea surface temperature [29, 30, 31].

Genetic analysis of the Chilean populations of the isopod *Excirolana braziliensis* Richardson, 1912 found three geographically separated groups related to its distribution, with strong evidence they are three different cryptic species [28]. A similar pattern is found for populations of the barnacle *Notochthamalus scabrosus* (Darwin, 1854), which has a restricted gene flow between populations from North and South of the barrier. The confamilial species *Jehlius cirratus* (Darwin, 1854), however, has only a marginal signal for isolation by distance [25]. This different pattern among similar barnacles might be a result of different larval behavior in the water column [32, 33, 25].

Our main goal is to understand how meiofaunal populations are connected along the Chilean Coast and if the biogeographical break plays a role in the genetic variability of meiofauna individuals. To address this, we analyze aspects of the phylogeography and population genetics of two morphotypes of *Ototyphlonemertes* found at eight localities along the Chilean coast: an *Ototyphlonemertes* Lactea morph and an *Ototyphlonemertes* Pallida morph. This is the first population genetics assessment for the Chilean meiofauna.

## Materials and methods

### Sampling and DNA sequence

**Fig 1:**
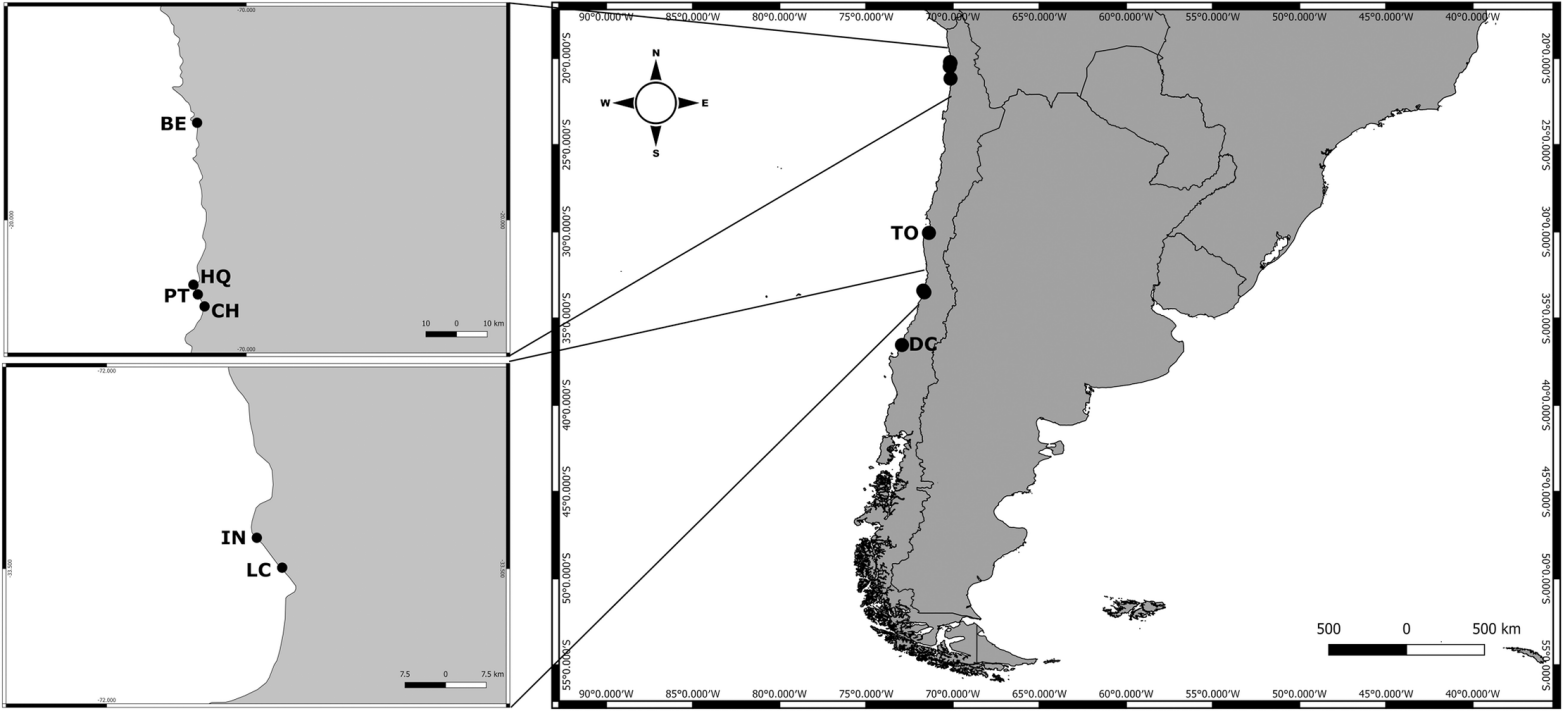
Sampling sites and coordinates along the Chilean coast. BE, Bellavista; HQ, Huaiquique; PT, Pozo Toyo; CH, Chipana; TO, Totoralillo; IN, Isla Negra; LC, Las Cruces; DC, Dichato.

One hundred and sixty-two individuals of Lactea and 25 individuals of Pallida were collected from sand samples in the intertidal zone following Corrêa’s [21] protocol. The animals were identified morphologically following Envall and Norenburg [23] by: (1) color of epidermis and brain region; (2) presence and position of the cephalic furrow; (3) presence and shape of cerebral organs; (4) proboscis papillae; (5) diaphragm size; (6) position and number of the accessory stylet pouches; (7) stylet structure; (8) shape of middle chamber; and (9) statolith type. The worms were collected in 2006, from eight sites along the Chilean coast: Chipana, Bellavista, Pozo Toyo, Huayquique (Tarapacá region), Totoralillo (Coquimbo region), Isla Negra, Las Cruces (Valparaíso region) and Dichato (Biobío region), with Lactea found at all eight and Pallida found at five of these sites (Fig 1 and Table 1). Living individuals were identified in the field utilizing a stereo and a compound microscope and preserved in ethanol 95% and, subsequent to field work, kept at -20°C until the DNA extraction. No permits were required for collecting meiofauna in Chile in 2006. All specimens were collected and transported in accordance with laws and permits.

**Table 1:** Collect sites of Lactea and Pallida morphotypes.

Locality	Region	Latitude	Longitude	Lactea	Pallida
Bellavista	Tarapacá (I)	20° 13’43” S	70° 09’21” W	*	
Huayquique	Tarapacá (I)	20° 16’42” S	70°07’50” W	*	*
Pozo Toyo	Tarapacá (I)	20° 29’01” S	70° 10’37” W	*	*
Chipana	Tarapacá (I)	21°10’43” S	70° 07’50” W	*	
Totoralillo	Coquimbo (IV)	30°04’26” S	71°22’31” W	*	*
Isla Negra	Valparaíso (V)	33° 24’10” S	71° 42’07” W	*	*
Las Cruces	Valparaíso (V)	33° 30’08” S	71° 38’09” W	*	
Dichato	Biobío (VIII)	36°32’26” S	72° 56’02” W	*	

* indicates presence.

Genomic DNA was extracted using the CTAB method described by Thollesson [34]. Partial regions of mitochondrial cytochrome *c* oxidase subunits 1 and 3 genes (hereafter COI and COX3) were amplified using the primer pairs LCO1490 ⁄ HCO2198 [35] for COI and COX3F/COX3R for COX3 (50-TGCGWTGAGGWATAATTTTATTTATT-30 and 50-ACCAAGCAGC TGCTTCAAAACCAAA-30, respectively) [36]. Polymerase chain reactions (PCR) were performed as follows: initial denaturation at 94°C for 1 min; 31 cycles of denaturation at 94°C for 30 s; annealing at 43°–48°C for 1 min, extension at 72°C for 1 min; and final extension at 72°C for 3 min. PCR products were purified with ExoSAP-IT (USB). Sequencing reactions for both strands of amplified markers were run using BigDye Terminator Cycle Sequencing Kit v3.1 (Applied Biosystems) and the amplification primers. Products were cleaned using Sephadex columns, dried, and sequenced using an Applied Biosystems automated sequencer. The products were sequenced using CEQ dye terminator chemistry and a CEQ 8000 Genetic Analysis System (Beckman Coulter, Brea, California). The sequence contigs were assembled using Sequencher, version 4.5 (Gene Codes, Ann Arbor, MI). Blast searches [37] were done on the NCBI website to check for possible contaminations. Sequences suspected of contamination or errors were removed. The analyses were performed with 128 and 100 Lactea individuals, and 19 and 9 Pallida individuals for COI and COX3, respectively (S1 Table). The final sequences are deposited in GenBank under the accession numbers MG 926401 –MG926542 for COI sequences and MG987308 –MG987418 for COX3 sequences.

### Data analysis

Sequences were aligned using MAFFT L-INS-i v.7 [38]. Phylogenetic trees were estimated with each morphotype partitioned for each marker in RAxML v8.2.9 [39] using the general time reversible model with gamma distribution (GTR+GAMMA). In order to evaluate the species relationships, phylogenetic trees were estimated with both markers concatenated and, because some specimens did not have sequences available for both markers, with each marker separately. This was a way to not lose information about any population. Sequences from *Nemertopsis tetraclitophila* Gibson, 1990 (GenBank accession number KF572482.1) were included as outgroup in the analysis for COI and COX3. In addition, a tree with COI sequences from this study and from Leasi *et al*. [20] (GenBank accession number KM083821.1—KM083889.1, KT730596.1—KT730625.1, KT722707.1—KT722730.1, KT730626.1—KT730678.1, KT736303.1—KT736314.1, KU230031.1—KU230131.1, KU230132.1—KU230244.1, KU230245.1—KU230291.1) was also estimated to evaluate the relationship between the Chilean species and other species of *Ototyphlonemertes*. A sequence from *Nipponemertes* sp. (GenBank accession number KU230295.1) was included as outgroup for this tree. All trees were estimated with 5,000 bootstraps replicates.

The resulting trees from RAxML analyses of single and concatenated markers were used as input to estimate species delimitation using the Bayesian implementation of the Poisson Tree Processes (bPTP software, available at the web-service http://species.h-its.org/) [40] with 5,000 MCMC generations, 100 thinnings and a burn-in of 0.1. Since bPTP analysis from COX3 did not converge, we also estimate delimitation using the General Mixed Yule Coalescent model (GMYC) [41]. Therefore, the sequences from COX3 were used to build an ultrametric tree in BEAST software v. 1.8.3 [42] with 600,000 MCMC generations, 100 thinnings and a burn-in of 6,000. This tree was used as input to GMYC. The COI sequences also were used in the web server of Automatic Barcode Gap Discovery for primary species delimitation software (ABGD, http://wwwabi.snv.jussieu.fr/public/abgd/abgdweb.html) [43] with default conditions, to complement the species delimitation analyses. Haplotype networks of both genes singly and concatenated were assessed using statistical parsimony [44], as implemented in the program TCS v1.21 [45]. The program connects haplotypes with the smallest number of differences as defined by 95% confidence criterion or the user can set the maximum number of accepted differences for connection between haplotypes. Here we set the maximum number of differences to 350, due to the highly polymorphic sequences. Results from the species delimitation analyses and TCS were compared to delimit possible cryptic species. All analyses performed after the species delimitation were in accord with these results. Therefore, the populations considered here are the ones delimited in such analyses.

The population analyses were performed with Arlequin v3.5 software [46]. The parameters used to describe genetic diversity at each locality and each marker were the number of polymorphic sites (*S*), haplotype diversity (*h*) [47], the nucleotide diversity (*π*) [48], and the mean number of pairwise differences (*k*) [49]. Nucleotide diversity (*π*) and haplotype diversity (*h*) were tested for significant differences among the groups by a non-parametric Kruskal-Walis in R software [50]. To infer whether populations went through recent expansion, the mismatch distribution from each locality was calculated in Arlequin with 1,000 permutations. In addition, to test recent population expansion, Tajima’s D [48] and Fu’s Fs [51], were also calculated, with 1,000 permutations.

To infer the degree of genetic differentiation within and between the individuals (from same locality and group), as well as between the different groups (possible cryptic species found in the species delimitation analyses), AMOVA [52] was performed in Arlequin with 20,000 permutations. In addition, a permutation test of genetic differentiation based on a nearest-neighbor statistic (Snn) [53] was conducted in DNAsp v5 [54] with 10,000 randomizations and an alpha value adjusted by Bonferroni correction for multiple tests.

The relationship between the genetic distance (Φ_st_ values) [52] and geographic distance (calculated as the minimum distance in kilometers between the two localities) was evaluated using Reduced Major Axis (RMA) regression. The correlation between the two distances was assessed by the Mantel test [55], implemented in the isolation-by-distance software (IBDWS v.3.23: http://ibdws.sdsu.edu/~ibdws/) [56], with 20,000 randomizations. Long-term gene flow inference was estimated by Migrate-n v3.6 software [57]. Following a burn-in of 20,000, 2.0 x 10^6^ genealogies were recorded at a sampling increment of 200 iterations. An adaptive heating scheme using four simultaneous Markov chains was implemented to increase the efficiency of searches. The same settings were used for all the groups. The long-term gene flow estimate M was converted to the average number of migrants per generation (*xNm*), using the formula *xNm* = *θ*_*i*_ x *M*_*i→j*_. Two separated runs were performed and the *xNm* from each run was used to calculate a median. A non-parametric Kruskal-Wallis was performed in R [50] to assess the significance of the number of migrants per generation toward north and south. Also, a pairwise Student’s *t* was used to compare the number of migrants sent and received by each locality.

## Results

### Haplotype networks and species delimitation

To avoid biasing or misdirecting the results we used only sequences with no ambiguities, which included trimming an average of 100 bp from each end of each gene fragment, resulting in final sequences of 605 bp and 362 bp for COI and COX3 respectively.

**Fig 2:**
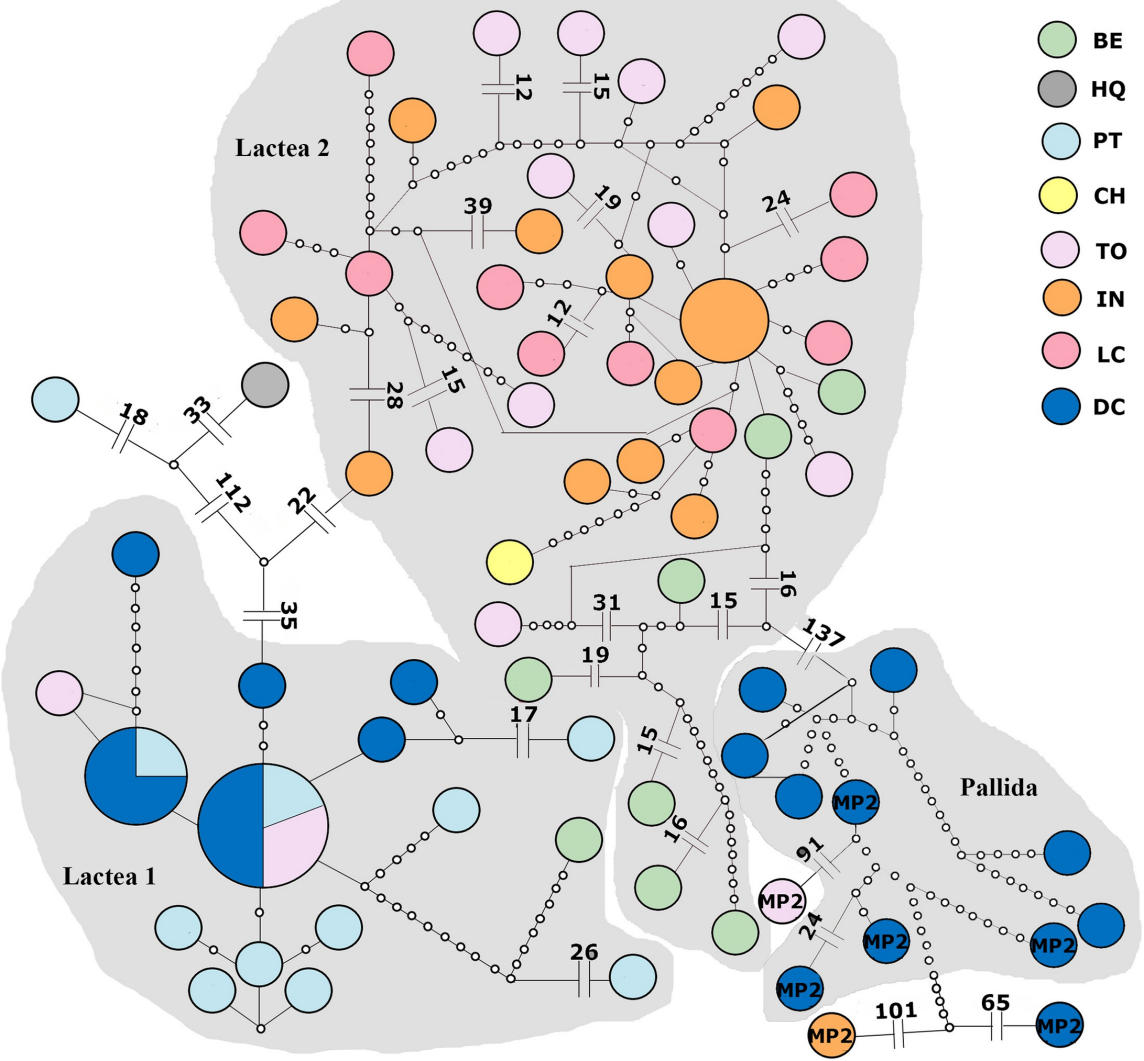
Haplotype network from concatenated genes (COI and COX3), showing three groups. Lactea 1, Lactea 2 and Pallida (recovered from bPTP and TCS analysis). Pallida morphotype haplotypes are identified, the remaining are Lactea morphotype. Locality abbreviations as in Fig 1.

The species delimitation analyses for all specimens and concatenated genes (81 individuals) sorted the individuals into three major groups: two Lactea (Lactea 1, Lactea 2) and one Pallida, with support values above 50, plus seven singletons (S1 Fig). The same pattern is seen in the haplotype networks from these individuals (Fig 2). The two groups formed by Lactea individuals, Lactea 1 and Lactea 2, are 58 mutational steps apart from each other, and Pallida is 137 mutational steps from Lactea 2. It should be noted here that a group of ten Pallida individuals from DC evidently were mistakenly recorded in the field as Lactea and, on the strength of an abundance of COI sequence data for the genus in Genbank, are treated here as Pallida.

**Fig 3:**
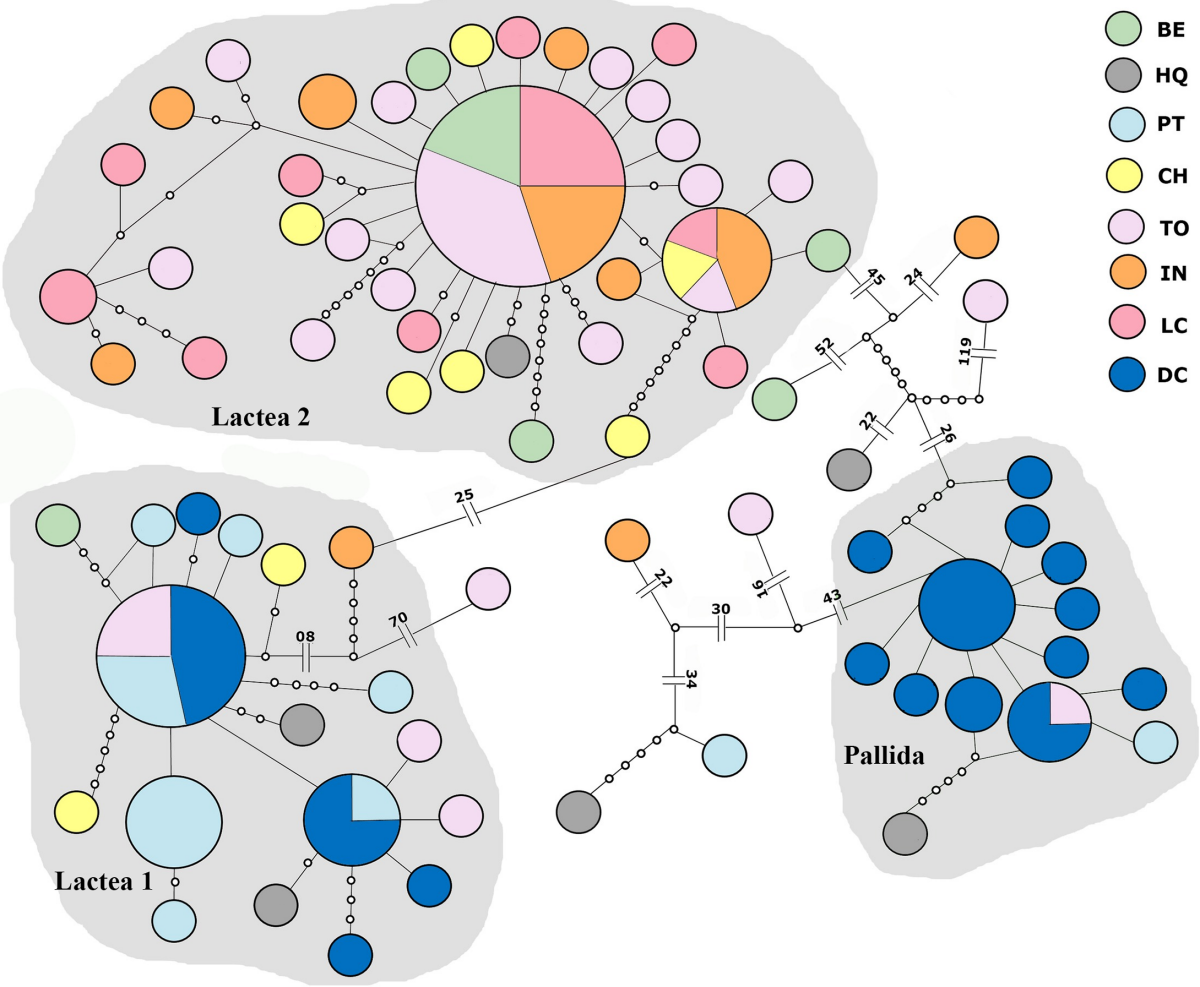
Haplotype network from COI sequences, showing the two Lactea and one Pallida groups recovered from bPTP and TCS analysis. Locality abbreviations as in Fig 1.

The species delimitation analyses with only COI sequences (147 individuals) has the same pattern as the concatenated analysis, with three major groups, but the number of singletons varies depending on the software used. With bPTP analysis, which gave the results with highest supports, there are 9 singletons (S2 Fig). In ABGD analysis there are 10 singletons and a fourth group consisting of two individuals (data not shown). GMYC allocates all individuals into three groups plus one singleton, but presents low values of support for the three groups (results not shown). The three groups are also recovered in the haplotypes networks from COI sequences (Fig 3). This network presents 68 haplotypes and 301 polymorphic sites with most of the haplotypes exclusive. Lactea 1 and Lactea 2 are separated by 25 mutational steps, while Lactea 2 and Pallida are 78 mutational steps apart. The singletons from bPTP are also excluded from the groups formed in the haplotype networks, some by large numbers of mutational steps. Therefore, those nine specimens were discarded from further analyses.

**Fig 4:**
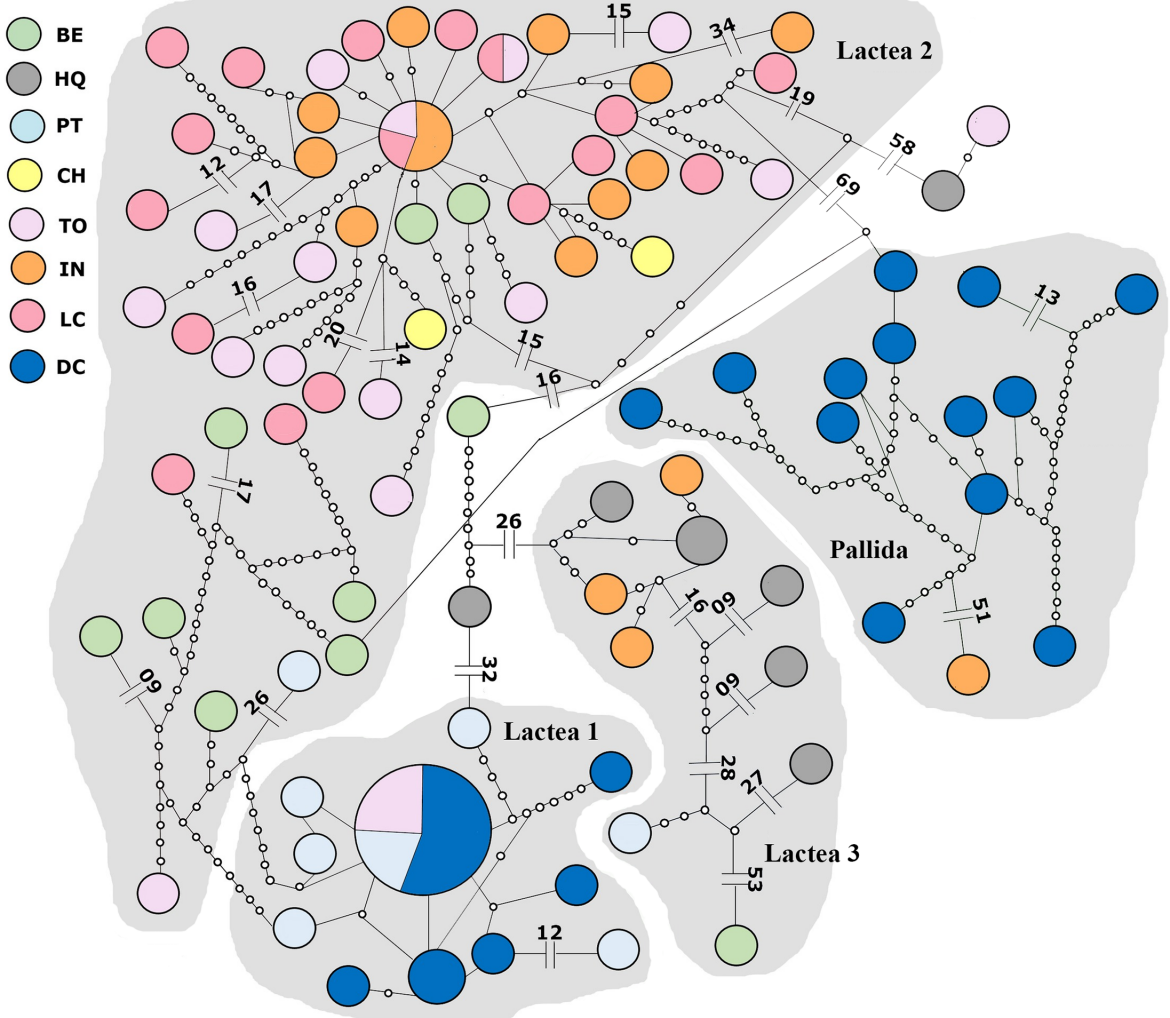
Haplotype network from COX3 sequences, showing Lactea 1, 2, and 3, and Pallida. Locality abbreviations as in Fig 1.

GMYC results from COX3 sequences (109 individuals) subdivide Lactea 1 and Lactea 2 in two groups each, but Pallida remains as one group (S3 Fig). In this analysis a fourth group, Lactea 3, is also delimited with individuals of Lactea morphotype represented only by COX3 sequences. The haplotype network presents 91 haplotypes with 566 polymorphic sites. This network shows the four groups as separate entities where Lactea 1 is 82 mutational steps apart from Lactea 2, Lactea 2 is 72 steps apart from Pallida and 76 apart from Lactea 3 (Fig 4).

The results from species delimitation analyses from concatenated and single markers were compared with each other and with the network haplotypes. From these comparisons, we are able to separate two morphological species as four different groups.

**Fig 5:**
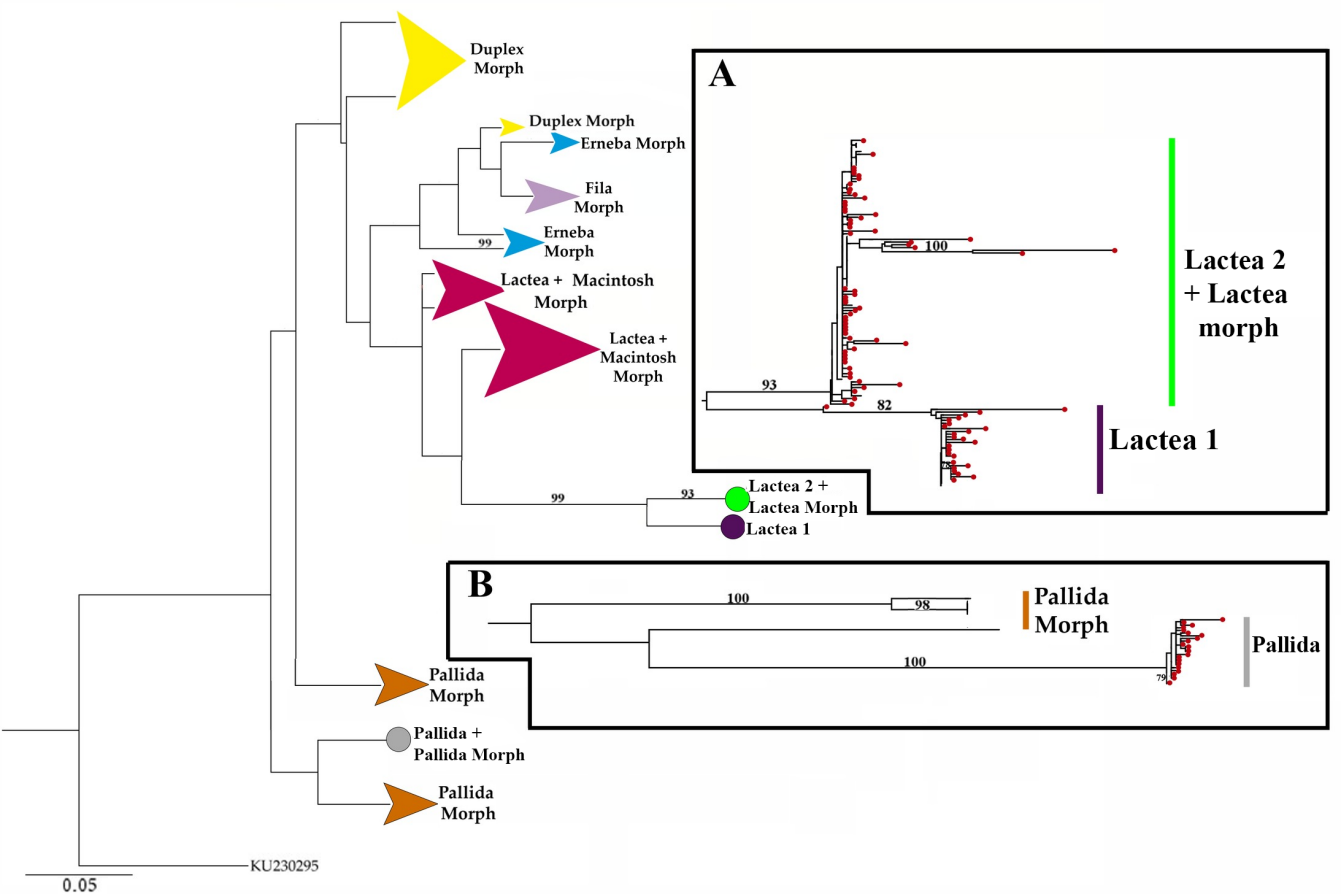
Maximum likelihood tree with specimens from Chile and from Leasi *et al*. 2016. **(A)** Lactea groups. **(B)** Zoom showing the clade formed with specimens of Pallida. Terminals with individuals from Chile are red. Bootstrap values > 70 noted above each branch (InL: -16353.45).

The ML analyses (lnL: -16353.45) for all COI sequenced specimens is presented in Fig 5. The phylogenetic tree with all *Ototyphlonemertes* placed Lactea 1 and Lactea 2 into two separate clades and with the Lactea morphotype individuals of Leasi *et al*. [20] also collected in Chile. Pallida individuals come out closely related to a Pallida group from Belize (Fig 5B) of Leasi *et al*. [20].

### Morphology

It should be noted here, *inter alia*, that this Chilean Pallida morphotype, with well-developed cerebral organs, a long, stout proboscis and its armature, is consistent with characteristics of the Pallida morphotype described by Envall and Norenburg [23] and could correspond to *Ototyphlonemertes santacruzensis* Mock and Schmidt, 1975, described from the Galapagos, for which there is no available genetic data for a morphologically confirmed specimen. The only field photomicrographs of one of our Chilean Pallida shows a pair of statocysts each with an irregular cluster of about six statolith granules (S3 Fig), rather than the four granules reported for *O*. *santacruzensis* and common for Pallida morphs found elsewhere in the world (JLN, pers obs). It is not unusual to see supernumerary granules in Pallida morphs but unusual to see almost identical irregularity in both statocysts (*ibid*.). *Ototyphlonemertes cirrula* Mock and Schmidt, 1975, also described from the Galapagos, resembles generally a Pallida morph but differs from all other *Ototyphlonemertes* in possessing epidermal cirri along the entire body length (these are easily overlooked by an inexperienced collector). More critically, the single *O*. *cirrula* described by Mock and Schmidt [22] is reported to have statocysts with up to 20 granules. However, one of us (JLN) found four individuals of *Ototyphlonemertes* on the Pacific coast of Panama with cirri along the entire body length but with statocysts having 6–8 granules, much as in S3 Fig, but we lack genetic data for these specimens. Hence, we need more morphological observations for the Chilean individuals, as well as morphological and molecular observations for those from the Galapagos.

Similarly, we have field photomicrographs for only a single Chilean Lactea. These reveal morphological affinity with *Ototyphlonemertes lactea* Corrêa, 1955 from Brazil, but also with *Ototyphlonemertes americana* Gerner, 1969 as reported from the Galapagos Islands by Mock and Schmidt [22]. All lack cerebral organs and cephalic cirri, have a very short proboscis with a thin irregular stylet basis, accessory stylets next to the basis, and a short cylindrical proboscis mid-bulb. However, the mid-bulb in our specimen (S3 Fig) is slightly more elongate than depicted for the other two species but not as long as is characteristic for the Macintoshi morphotype [23].

### Genetic diversity and gene flow estimates

To avoid causing an artifact by mixing probable different evolutionary units, the four evolutionary units were analyzed separately for intra- and interpopulational parameters. All localities from each group show moderate to high haplotype diversity (Table 2). There is no statistically significant difference in haplotype diversity between markers in Lactea 1 and Lactea 2, but COX3 values are higher and statistically different in Lactea 2 (Kruskal-Wallis *x*^*2*^ = 5.54, *p* = 0.0186). There also is no statistically significant difference among groups for COI or COX3. The nucleotide diversity is very variable, with no statistically significant difference between markers. Likewise, values of nucleotide diversity are not statistically different between groups for COI or COX3. The average pairwise differences (*k*) show high intra-population variation, with no statistically significant difference between markers or between groups in both COI and COX3 (Table 2).

**Table 2:** Diversity indexes for COI and COX3 for Lactea and Pallida morphotypes.

**Groups**	**Samples(n)**	***H***	***S***	***h***	***π*(SD)**	***k*(SD)**
Lactea 1	BE (1)	1	0	-	-	-
	PT (15)	9	15	0.879	0.006(0.004)	3.6(1.9)
	HQ (2)	2	9	1.000	0.016(0.017)	9(6.7)
	CH (2)	2	27	1.000	0.050(0.050)	28.0(20.1)
	TO (6)	3	5	0.600	0.003(0.002)	1.9(1.2)
	DC (14)	5	9	0.802	0.003(0.002)	2.0(1.2)
Lactea 2	BE (9)	4	18	0.722	0.008(0.005)	4.9(2.6)
	HQ (1)	1	0	-	-	-
	CH (6)	6	35	1.000	0.026(0.016)	13.9(7.3)
	TO (24)	11	70	0.798	0.015(0.008)	8.8(4.2)
	IN (17)	8	75	0.875	0.19(0.01)	11.5(5.5)
	LC (17)	9	23	0.899	0.007(0.004)	4.1(2.2)
Pallida	HQ (1)	1	0	-	-	-
	PT (2)	2	0	1.000	-	-
	TO (1)	1	0	-	-	-
	DC (20)	11	18	0.914	0.005(0.003)	2.8(1.6)
**Groups**	**Samples(n)**	***H***	***S***	***h***	***π*(SD)**	***k*(SD)**
Lactea 1	BE (1)	1	0	-	-	-
	PT (10)	8	55	0.933	0.035(0.019)	12.2(6.1)
	TO (5)	5	19	1.000	0.022(0.014)	7.6(4.3)
	DC (14)	14	10	1.000	0.005(0.003)	1.9(1.1)
Lactea 2	BE (7)	7	79	1.000	0.099(0.056)	34.8(17.3)
	CH (2)	2	12	1.000	0.034(0.035)	12(8.8)
	TO (13)	13	79	1.000	0.051(0.027)	18.1(8.6)
	IN (13)	13	52	1.000	0.027(0.015)	9.4(4.6)
	LC (17)	17	108	1.000	0.066(0.034)	23.4(10.8)
Lactea 3	BE (1)	1	0	-	-	-
	HQ (6)	5	91	0.933	0.107(0.062)	37.8(19.3)
	PT (1)	1	0	-	-	-
	IN (3)	3	7	1.000	0.013(0.011)	4.67(3.1)
Pallida	TO (1)	1	0	-	-	-
	IN (1)	1	0	-	-	-
	DC (13)	13	54	1.000	0.051(0.027)	18.3(8.7)

n–number of individuals; H–number of haplotypes; S–number of polymorphic sites; h–haplotype diversity; π –nucleotide diversity; k–average number of pairwise differences; SD–standard deviation.

Table 3 presents the AMOVA results for comparisons within and among localities for both markers. Most of the variability is within the localities in each group for both regions, except for Pallida. The pairwise Φ_st_ values show signs of differentiation between some populations in all groups (S4 Fig), but these signs don’t have a geographic pattern. In Lactea 1 for example, CH show the same degree of differentiation for both PT (a north population) and TO (a south population). Lactea 2 has low values of differentiation between all populations but HQ has high Φ_st_ values relative to BE, TO and LC. In Pallida and Lactea 3, due to the high intra-population variation, most of populations show high values of differentiation (S4 Fig).

**Table 3:** AMOVA and Snn results for COI and COX3 in Lactea and Pallida morphotypes.

**Group**	**Hierarchical level (*df*)**	**Variance**	**% Total**	**Φ**_**ST**_	**Snn**
**Lactea 1**	Among populations (5)	0.720	30.39	0.30*	0.41***
	Within populations (33)	1.649	69.61		
**Lactea 2**	Among populations (5)	0.229	5.39	0.053*	0.28*
	Within populations (68)	4.019	94.61		
**Pallida**	Among populations (3)	1.244	48.89	0.49*	1.00
	Within populations (20)	1.300	51.11		
**All populations**	Among groups (2)	39.330	91.62	0.93***	0.44***
**Group**	**Hierarchical level (*df*)**	**Variance**	**% Total**	**Φ**_**ST**_	**Snn**
**Lactea 1**	Among populations (3)	0.526	14.21	0.14	0.46*
	Within populations (26)	3.174	85.79		
**Lactea 2**	Among populations (4)	2.500	20.28	0.20***	0.21
	Within populations (47)	9.830	79.72		
**Lactea 3**	Among populations (3)	10.239	41.95	0.42*	0.59
	Within populations (7)	14.167	58.05		
**Pallida**	Among populations (2)	32.063	77.82	0.78**	1.00***
	Within populations (12)	9.141	22.18		
**All populations**	Among groups (3)	21.593	63.13	0.76***	0.42***

df—degrees of freedom; Snn–nearest-neighbour statistic

* p < 0.05

** p < 0.01

*** p < 0.001

Results from Tajima’s D analysis show most localities in each group are in equilibrium between mutation and genetic drift, with no evidence of recent geographic expansion. However, the results show evidence of geographic expansion and signs of recovery from a recent bottleneck in seven localities for COI region, and three for COX3 (Table 4). The Fu’s FS, on the other hand, shows only two localities in recent geographic expansion for COX3 and one, Pallida_DC, with conflicting results for each marker. In COI analyses, the localities show signs of population retraction and in COX3 show signs of population expansion (Table 4). Mismatch results shows signs of population expansion only in one locality, Lactea 1_DC. All other localities present no signs of expansion or retraction.

**Table 4:** Neutrality tests and mismatch distribution results from COI and COX3 for Lactea and Pallida morphotypes.

				***Mismatch***			
**Groups**	**Localities**	***D***	***FS***	***Rgd***	***t***	***θ***_***0***_	***θ***_***1***_
**Lactea 1**	BE	0	0	-	-	-	-
	HQ	0	2.19	-	-	-	-
	PT	1.62*	-1.41	0.01	1.45	2.71	8.59
	CH	0	3.33	-	-	-	-
	TO	-0.82	0.89	0.52	4.64	0	2.74
	DC	-1.57*	-2.14	0.05*	0.88	0	99.99
**Lactea 2**	BE	-1.93***	1.15	0.07	0	0	99.99
	HQ	0	0	-	-	-	-
	CH	-1.02	-0.47	0.13	6.39	0	574.37
	TO	-2.17**	-1.92	0.01	0.12	0	99.99
	IN	-2.24***	1.08	0.02	0.56	0	99.99
	LC	-1.64*	-2.20	0.02	4.34	0.01	6.87
**Pallida**	HQ	0	0	-	-	-	-
	PT	0	0	-	-	-	-
	TO	0	0	-	-	-	-
	DC	-1.74*	7.15***	0.04	2.7	0	99.99
				***Mismatch***			
**Groups**	**Localities**	***D***	***FS***	***Rgd***	***t***	***θ***_***0***_	***𐎸***_***1***_
**Lactea 1**	BE	0	0	-	-	-	-
	PT	-1.86**	0.13	0.72	0.56	6.52	99.99
	TO	-1.22*	6.04	0.68	3	0	0.41
	DC	-1.5	1.03	0.07	0.04	0	99.999
**Lactea 2**	BE	0.41	0.26	0.06	16.7	31	1005.79
	CH	0	2.48	-	-	-	-
	TO	-1.30	-3.53*	0.02	11.3	9.97	317.65
	IN	-1.97*	-2.23	0.02	4.69	0	26.80
	LC	-1.17	-4.78*	0.01	1.99	18.6	99.99
**Lactea 3**	BE	0	0	-	-	-	-
	HQ	-0.33	3.08	0.20	20.5	5.61	945.63
	PT	0	0	-	-	-	-
	IN	0	0.30	0.66	5.25	0	99.99
**Pallida**	TO	0	0	-	-	-	-
	IN	0	0	-	-	-	-
	DC	-0.20	-3.50*	0.03	6.96	15.8	3,74

D—Tajima's D results; FS—Fu's Fs results; Rgd—Harpending's Raggedness index; t–units of divergence time; θ_0_ –function of population size before expansion; θ_1_ –function of population size after expansion. Locality abbreviations as in Fig 1.

* p < 0.05

** p <0.01

*** p < 0.001

Results from Mantel tests show no statistically significant relationship between geographic and genetic distance in Pallida for COI and in all groups for COX3. Lactea 1 and Lactea 2 show a positive relationship between geographic and genetic distance for COI (*r* = 0.622, *p* = 0.0031, and *r* = 0.025, *p* = 0.0404, respectively), despite co-occuring in some localities.

Migrate-n results are shown in Tables 5 and 6. Pallida results are discordant between the two markers. However, this group is formed by individuals from different localities in COX3 and COI analyses. In general, all populations have high rates of migrants, with the exception of BE in Lactea 1 and all populations of Pallida when COI is analyzed. These results also show no statistical difference for migrant exchange between north and south populations for any group.

**Table 5:** Migrate-n results from COI for Lactea and Pallida morphotypes.

COI							
Groups	Receiver/ Sender	BE (<HQ^1^, PT^2^ & CH^3^)	HQ	PT	CH	TO	DC (<CH)
**Lactea 1**	**BE**		46.70 (1.7–66.1)	1.95 (0–96.2)	93.09 (2.6–123)	0.033 (0–67.3)	3.52 (3.4–69.4)
	**HQ**	0.06 (0–8.6)		52.26 (0–108)	55.65 (3.5–98.3)	32.19 (2.3–67.9)	16.45 (0.4–62.6)
	**PT**	0.19 (0–9.3)	49.24 (4.1–67.8)		33.03 (0.6–68)	30.91 (3.8–67.6)	15.53 (0.3–59.3)
	**CH**	0.03 (0–8.4)	34.54 (2.4–65.9)	46.50 (5.7–95.7)		45.77 (3.9–67.9)	10.74 (0–57.9)
	**TO**	0.66 (0–8.6)	2.29 (0–16.2)	4.69 (0.6–82)	53.08 (4.7–120.5)		2.60 (0.9–18.2)
	**DC**	0.07 (0–5.2)	48.11 (1.8–66.5)	87.79 (0–126)	50.50 (1.3–88.7)	30.21 (3.8–69)	
		**BE**	**HQ**	**CH**	**TO** **(>BE**^**4**^ **& IN**^**5**^**)**	**IN**	**LC**
**Lactea 2**	**BE**		37.69 (10–70.1)	66.32 (9.2–128.4)	56.28 (18–129.2)	35.07 (1.8–68)	66.25 (14.2–120.6)
	**HQ**	55.97 (12.6–68.8)		49.24 (9.2–116)	103.12 (35.1–130)	57.25 (11.9–68.3)	114.71 (39.7–131)
	**CH**	33.72 (9.1–59.6)	60.20 (17.2–96.4)		84.52 (16.6–126.1)	7.26 (0.1–28.1)	99.24 (23.2–128.6)
	**TO**	56.69 (16.7–69)	56.92 (15.5–195)	111.47 (18.6–130)		43.25 (9.9–69.2)	66.57 (13.3–88.1)
	**IN**	13.32 (0.8–35.4)	13.44 (1–39.3)	68.34 (15.6–93.5)	69.83 (24–106.7)		7.05 (0.1–26.7)
	**LC**	13.90 (2.2–58.8)	73.36 (16–92.1)	83.84 (16.1–109)	113.16 (39–131.7)	18.76 (2.7–60.7)	
		**HQ**	**PT** **(<HQ**^**6**^ **& DC**^**7**^**)**	**TO** **(<DC**^**8**^**)**	**DC**		
**Pallida**	**HQ**		0.002 (0–14.3)	0.066 (0–12.6)	56.52 (1.8–104.3)		
	**PT**	1.02 (0–10)		0.066 (0–12.7)	2.69 (0–113.1)		
	**TO**	1.80 (0–20.5)	0.006 (0–12.2)		19.22 (0.7–116.7)		
	**DC**	46.44 (0.4–63.1)	0.0001 (0–12.2)	0.008 (0–11.7)			

Localities within parenthesis received a statistically different number of migrants ('<' indicates that received more migrants; '>' indicates that received less migrants).

^1^*t* = -15.3, *p* = 0.007

^2^*t* = -14.9, *p* = 0.009

^3^*t* = -21.7, *p* = 0.0001

^4^*t* = -15, *p* = 0.04

^5^*t* = -14.8, *p* = 0.05

^6^*t* = 6.67, *p* = 0.003

^7^*t* = 8.33, *p* = 0.0007

^8^*t* = 5.33, *p* = 0.01.

Locality abbreviations as in Fig 1.

**Table 6:** Migrate-n results from COX3 for Lactea and Pallida morphotypes.

Groups	Receiver Sender	BE	PT	TO	DC	
**Lactea 1**	**BE**		105.02 (7.2–126.5)	10.01 (1.4–83.2)	0.38 (0–40.8)	
	**PT**	1.30 (0–85)		4.79 (0.1–73.6)	2.60 (0–75.2)	
	**TO**	38.82 (6–115.3)	36.03 (0.3–124.5)		4.64 (0–75.9)	
	**DC**	20.32 (0–64.8)	56.68 (4.5–70.7)	4.10 (0.7–66.6)		
		**BE**	**CH**	**TO**	**IN**	**LC**
**Lactea 2**	**BE**		38.65 (5.2–55.5)	9.81 (2.3–36.4)	13.91 (3.9–44.1)	43.42 (8.4–70.6)
	**CH**	17.36 (3–44.1)		0.48 (0–15.7)	35.36 (16.7–64.2)	55.28 (23.7–81.5)
	**TO**	47.40 (15.8–73.8)	23.64 (7.9–41)		49.43 (25.2–75.1)	66.61 (36.3–103.7)
	**IN**	14.44 (3.5–44.2)	19.45 (3.9–71.7)	44.14 (24.3–78.2)		42.97(13.2–67)
	**LC**	15.45 (2.9–30.8)	42.36 (11.3–74.8)	59.73 (35–87.3)	34.25 (10.9–56.1)	
		**BE**	**HQ**	**PT**	**IN**	
**Lactea 3**	**BE**		58.03 (3.1–85.4)	86.01 (5.7–125.9)	96.15 (8.7–129.2)	
	**HQ**	51.68 (6.5–105.5)		65.52 (3.5–119.5)	66.25 (3–118.2)	
	**PT**	63.67 (5.8–113.1)	61.56 (6.2–126.9)		30.84 (5–83.9)	
	**IN**	69.22 (9.4–126.7)	23.43 (0–102.4)	17.86 (1.6–83.8)		
		**TO**	**IN**	**DC**		
**Pallida**	**TO**		106.70 (28.9–129.8)	33.74 (10.1–65.6)		
	**IN**	52.35 (17.1–106.5)		78.22 (15.2–100.7)		
	**DC**	75.56 (30.8–124.4)	46.62 (6–103.2)			

Locality abbreviations as in Fig 1.

## Discussion

### Presence of cryptic species

The presence of cryptic species is a common feature of meiofaunal organisms, since most of these tiny animals have few morphological characters to be used in identification. Therefore, many meiofaunal species have been recorded as cosmopolitan despite their low dispersion ability. This pattern is known as the Meiofauna Paradox [2] and has been discussed in many studies (e.g., [2, 20, 58, 59, 60, 61, 62]). Molecular data has increasingly revealed that some of these supposedly cosmopolitan species are actually complexes of cryptic species with low dispersal ranges (*e*.*g*., [14, 19, 20, 59, 63, 64, 65]).

The presence of cryptic species in *Ototyphlonemertes* is something already observed in other molecular studies from the western Atlantic coast [17, 18]. Delimitation between species and ecological morphotypes is difficult [23, 24, 66] because morphological characters frequently overlap. There has never, however, been a case of overlap for two fundamental character differences between Lactea and Pallida morphs: respectively, spiral vs. smooth stylet, and lack of cerebral organs vs. presence. It is, however, possible with the tight travel schedule and modest field microscopy employed during collecting in this study, to mis-allocate individuals.

The morphological group Lactea has different morphotypes distributed worldwide. However, the anatomical features of these morphotypes are not enough to reliably separate those groups into different species [23]. The relationship between the cryptic species found here and previously characterized morphotypes is conditional on geographically more comprehensive study. Molecular data for all species described from the Galapagos not only could be very interesting for expanding this biogeographic study but they are needed to ascertain taxonomic affinities and status of the Chilean *Ototyphlonemertes*. The speciation might be caused by multiple colonization events causing genetic divergence and isolation in those groups [20], perhaps driven by ocean current, beach and weather dynamics.

The haplotype networks of both genes concatenated show a “parochial” pattern, where there is a high number of haplotypes, mostly restricted to one location. This pattern is characteristic of low-dispersal animals [67]. Despite this, dispersion probably occurred over long distances, since the haplotypes have few mutation steps between each other, though most of the haplotypes are geographically restricted. Haplotype networks analyzed for each group separately, reveals a different pattern for Lactea 2. This haplotype network has a more star-like pattern, with one dominant haplotype and many rare haplotypes differing by few mutations. Star-like networks are common in species with a strong founder effect; *e*.*g*., when there is re-colonization in areas where local extinction occurred [67].

The genetic diversity indexes, like nucleotide and haplotype diversity, are very high for both markers. Genetic diversity values can be high in populations where environmental conditions are stable for long periods at evolutionary scales, allowing for accumulation of genetic diversity [68, 69]. This might not be the case for *Ototyphlonemertes*, many of which live in disturbed environments with seemingly ephemeral populations, present in a location in some periods and completely disappearing at others [18, 20]. High values of nucleotide and haplotype diversity are also related to large populations over evolutionary times [67]. This most likely is the case for *Ototyphlonemertes* populations, since meiofaunal populations in general might occur in high numbers [70, 71, 72].

Neutrality tests show that most local groups are in equilibrium between mutation and genetic drift, with no expansion, but some have signs of recent expansion after a bottleneck. However, results from the three statistics used here (Tajima’s D, Fu’s Fs and Mismatch distribution) and for the two markers do not agree in some localities. The power of neutrality statistics decays with time since the expansion, but the pace is different for each test. For bottleneck events, Fu’s FS is the most powerful statistic; it, however, lacks power to detect bottlenecks that have just occurred. The same happens with population expansion and decrease, but Tajima’s D is also sensitive for population decrease [73]. According to Ramos-Onsis and Rozas [74], tests based on mismatch distribution are very insensitive to detecting population growth, requiring large samples and stronger changes to indicate population growth. Therefore, for small sample sizes, as in this study, Tajima’s D and Fu’s FS seem to be more powerful to detect population fluctuations. In this sense, despite the seasonal environment, *Ototyphlonemertes* local groups are in equilibrium, except for Pallida_DC, which seems to be under expansion in both Tajima’s D and Fu’s FS statistics for both markers. Therefore, while these populations seem to be ephemeral they might have mechanisms to survive nearby or rapidly recolonize these environments.

### Genetic structuring

Results from AMOVA and Migrate-n are congruent for most groups, with signs of gene flow in all groups, even between distant localities. Stochastic events, such as storms, were the mechanism suggested by Tulchinsky *et al*. [18] to explain long-distance dispersion found in *Ototyphlonemertes* along Florida and Caribbean coasts. Here the migration of these animals occurs most likely from larval [75] and occasional adult dispersal in ocean currents or by other dispersal mechanism (*e*.*g*., rafting–[76]). The Chilean coast has a turbulent tectonic history (earthquakes) and a complex system of ocean currents with superficial, subsuperficial and deep currents flowing northwards and southwards. The currents that flow near the coast are the Chile Coastal Current, the Chile Coastal Countercurrent, the Peru Current (Humboldt Current) and the Peru-Chile Undercurrent (Gunther Current) [77, 78]. The north-flowing Humboldt Current is the one that most influences the coastal communities between 18°S and 42°S [79].

Some studies report a biogeographic break between 30°S and 33°S based on the occurrence and distribution of benthic species, separating this coast into North and South (*e*.*g*., [26, 27, 77, 79, 80, 81, 82, 83, 84]). This historical discontinuity, which originated in the last glaciation, disrupted gene flow in the past but low-dispersal species still retain this genetic signature [85]. Species with high or medium dispersal do not present this genetic signature between populations from the North and South [85]. Although the species in *Ototyphlonemertes* genus are presumed, like other mesopsammic meiofauna [2], to have low dispersal capability [20, 23, 24], our results show no differentiation between species from North and South for the four groups studied here, which may be evidence of a greater dispersal potential than expected. In addition, the results of pairwise Φ_st_ (S5 Fig) showing low values of differentiation between the localities support this idea. Another possibility for this lack of differentiation is the short time between extinction and recolonization of these populations. The environment where these populations develop is variable, subject to sudden changes that can easily extinguish very localized populations and erase some genetic signals.

Lactea 1 and Lactea 2 show patterns of isolation by distance only for COI, with significant results from Mantel’s test, AMOVA and highly significant Snn values. The rate of migrants in these two groups supports the idea of connected populations, but since they are isolated by distance, it is probable that the connection occurs by stepping-stones [86]. For Lactea 2, comparing the results from both markers individually reveals that the local groups are connected, with a significant rate of migrants per generation between all localities. The Φ_st_ values are significant (S5 Fig) but low for mitochondrial markers, showing a level of structuring expected for populations with medium dispersion capability [67].

The different results found for the four groups further support the idea that these are four independent evolutionary entities with different ecological, dispersal and genetic characteristics. They each have most of their haplotypes geographically exclusive but the shape of the haplotype network is different for each group. In addition, Lactea 2 genetically is highly polymorphic, while Lactea 1, Lactea 3, and Pallida are less polymorphic, but still very diverse. The haplotype diversity is similar for all groups; the nucleotide diversity, however, is very low in Pallida, moderate in Lactea 1, and high in Lactea 2 and Lactea 3. Lactea 1 and Lactea 2 have significant isolation by distance, while Pallida and Lactea 3 do not. Also, the number of migrants per generation is very different between the groups. Therefore, these four groups do not present the same pattern in most of the characteristics analyzed, except for the lack of response to the historical biogeographic barrier present on the Chilean coast.

The results presented here suggest that these organisms exhibit patterns of gene flow similar to other organisms with high dispersal present in Chile, such as the isopods *Limnoria chilensis* and *Limnoria quadripunctata*, which have connected populations along the entire Chilean coast [85]. They, somehow, are capable of long distance dispersal in sufficient frequency to connect the populations along the Chilean coast but have complicated evolutionary histories. In keeping with the Meiofauna Paradox, we see here more evidence that meiofauna species can successfully conquer new environments through dispersal.

## Supporting information

S1 Table List of all specimens, their geographic localization and GenBank access numbers. Specimens from Leasi *et al*. 2016 are specified on the observations field.
(XLS)

S1 Fig Resulting tree of species delimitation analysis in bPTP with concatenated sequences. Numbers above the branches are the posterior probability of speciation events in each branch. Lactea 1 contains 24 specimens, Lactea 2 40 specimens and PallidaS 10 specimens.
(TIF)

S2 Fig Resulting tree of species delimitation analysis in bPTP with COI sequences. Numbers above the branches are the posterior probability of speciation events in each branch. Lactea 1 contains 24 specimens, Lactea 2 40 specimens and Pallida 15 specimens.
(TIF)

S3 Fig Resulting tree of species delimitation analysis in GMYC with COX3 sequences. The diagram in the upper left corner is the time at which the model infers that the threshold transition from the speciation-level events to the coalescent-level events takes place. Red branches were probably formed after the speciation events. Lactea 1 contains 29 specimens, Lactea 2 contains 53 specimens, Lactea 3 contains 11 specimens, and Pallida contains 15 specimens.
(TIF)

S4 Fig Photomicrographs of squeezed live specimens of Pallida and Lactea morphotypes. **(A)** Specimen of Pallida showing statocysts with six granules, marked with arrows. **(B)** Specimen of Lactea with everted proboscis showing the mid-bulb (MB).
(TIF)

S5 Fig Paired Φ_st_ values for COI and COX3 separated by groups and localities. Locality abbreviations as in Fig 1.
(TIF)
