# Supplementary figures and images for: Hidden diversity: Phylogeography of genus Ototyphlonemertes Diesing, 1863 (Ototyphlonemertidae: Hoplonemertea) reveals cryptic species and high diversity in Chilean populations
Source: PLoS One. 2018 Apr 26;13(4):e0195833. doi: 10.1371/journal.pone.0195833 (PMC5919620; doi:10.1371/journal.pone.0195833)

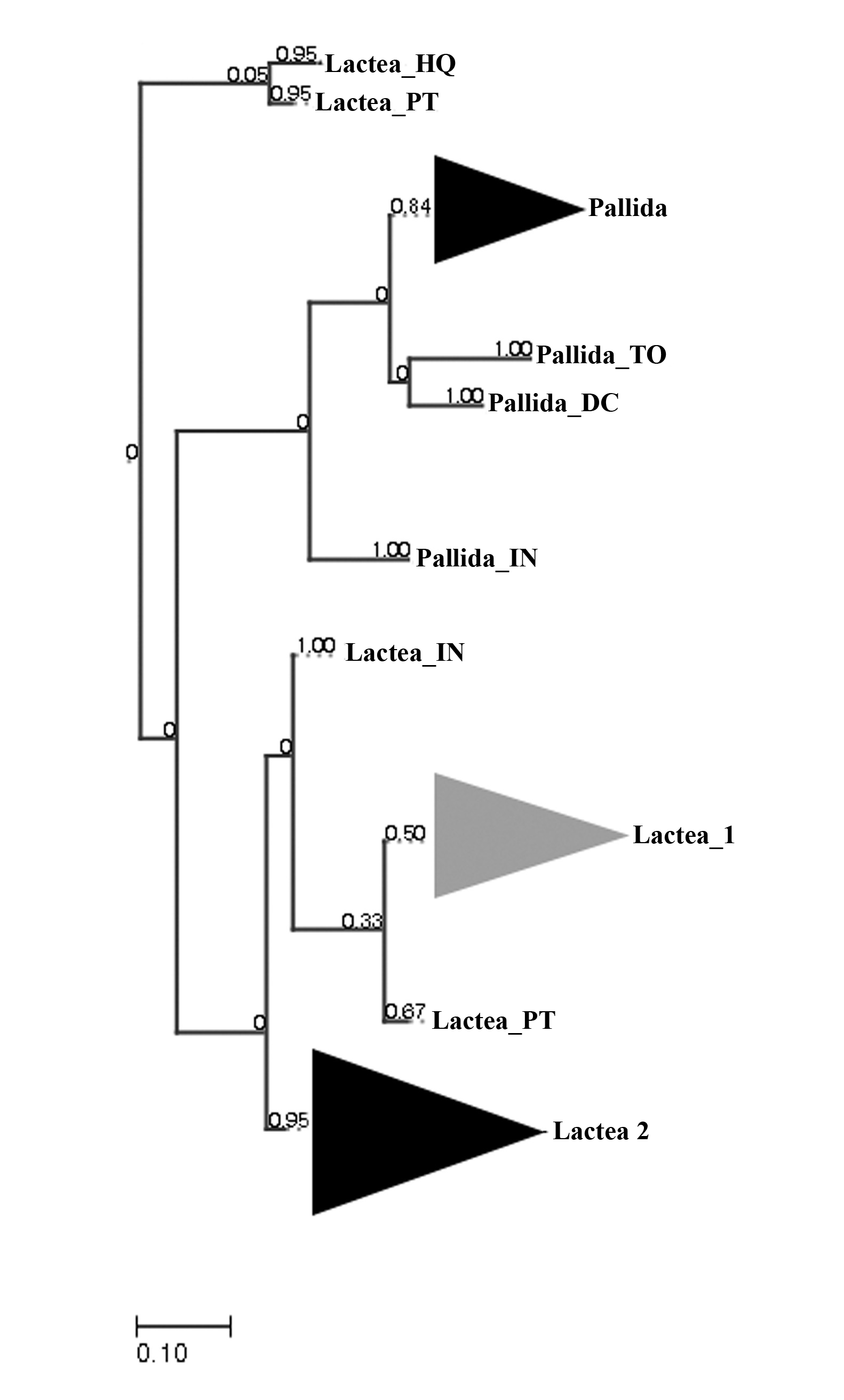

Supplement: S1 Fig — Numbers above the branches are the posterior probability of speciation events in each branch. Lactea 1 contains 24 specimens, Lactea 2 40 specimens and PallidaS 10 specimens. (TIF) [file pone.0195833.s002.tif]

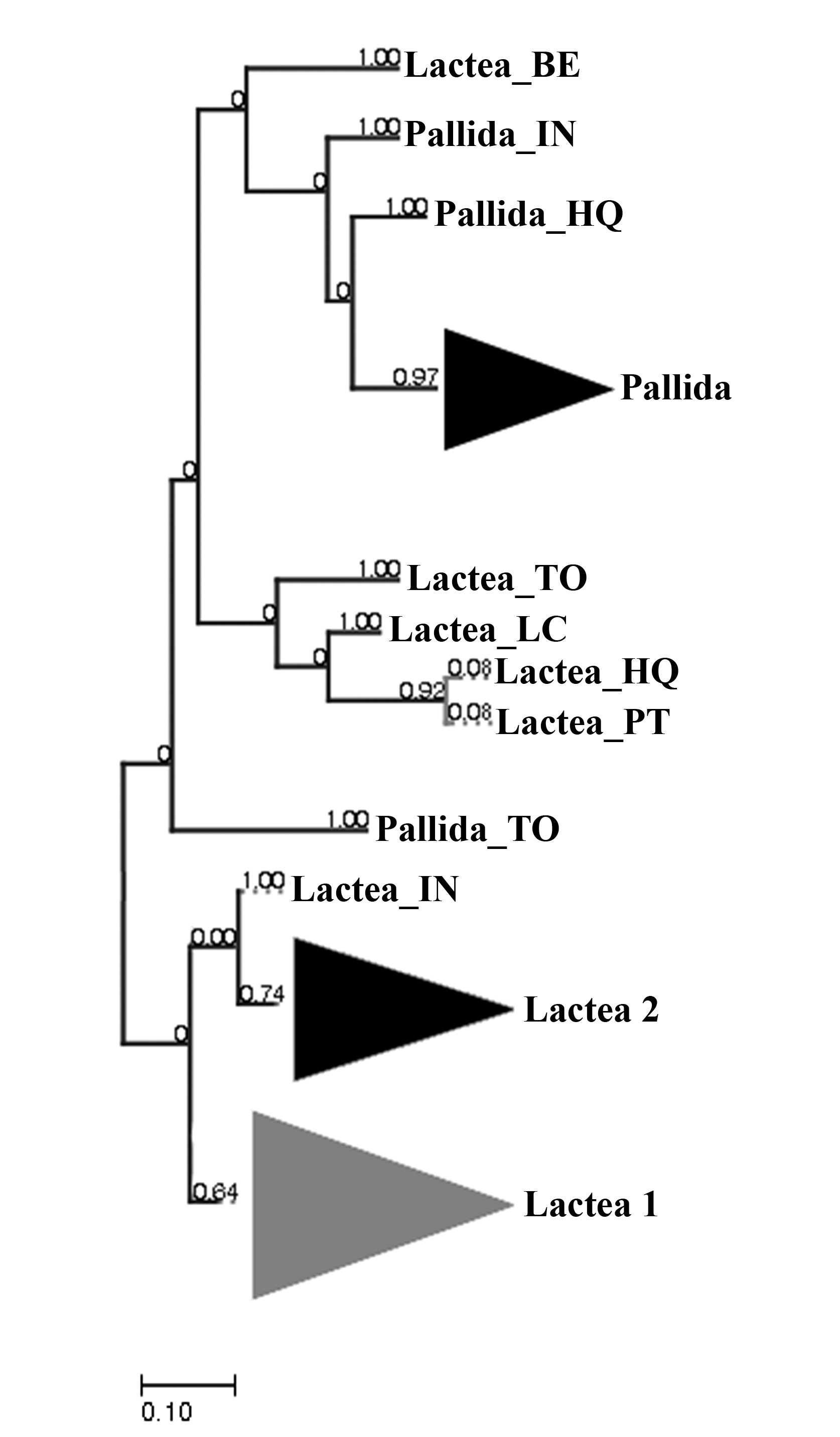

Supplement: S2 Fig — Numbers above the branches are the posterior probability of speciation events in each branch. Lactea 1 contains 24 specimens, Lactea 2 40 specimens and Pallida 15 specimens. (TIF) [file pone.0195833.s003.tif]

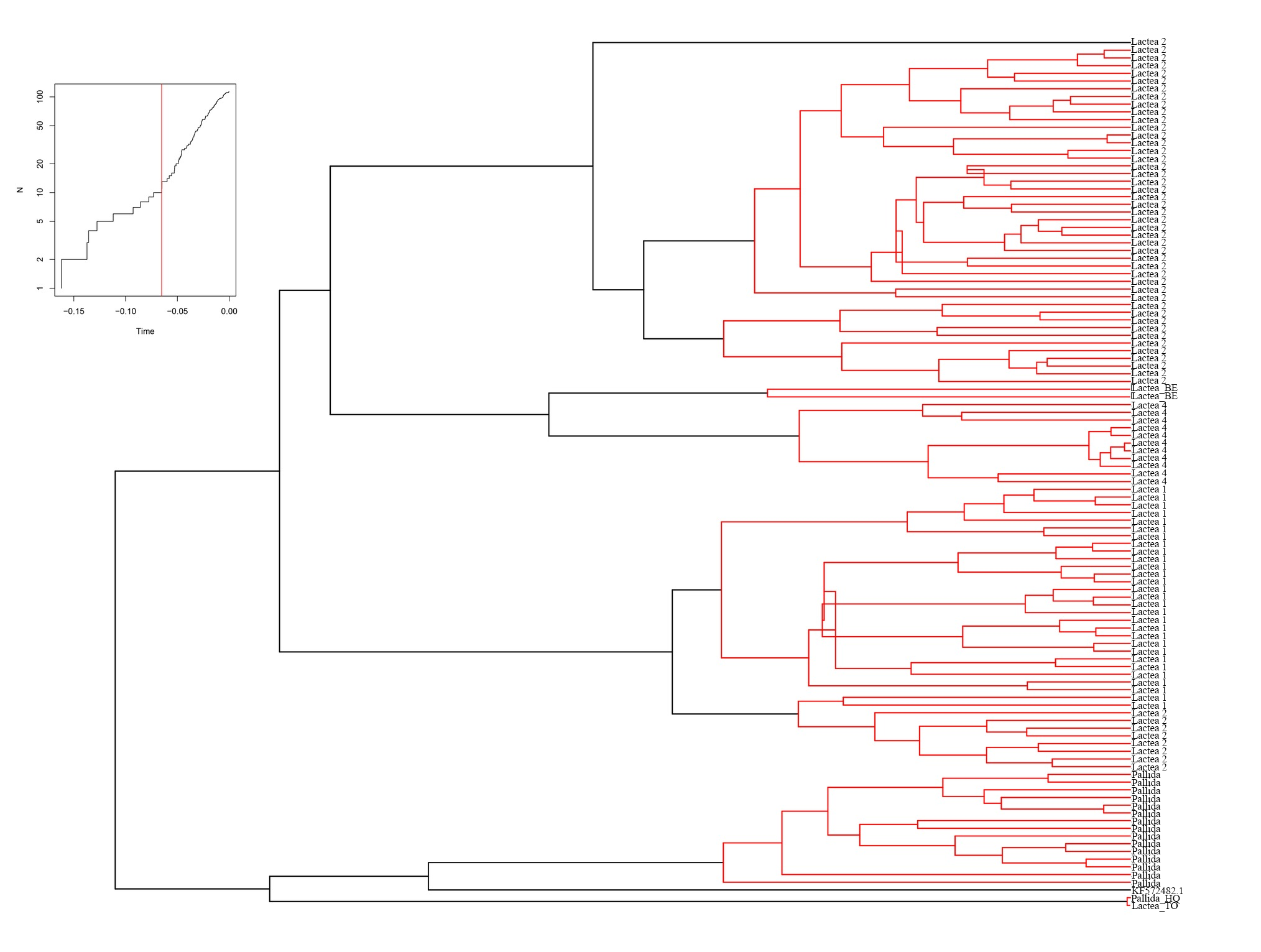

Supplement: S3 Fig — The diagram in the upper left corner is the time at which the model infers that the threshold transition from the speciation-level events to the coalescent-level events takes place. Red branches were probably formed after the speciation events. Lactea 1 contains 29 specimens, Lactea 2 contains 53 specimens, Lactea 3 contains 11 specimens, and Pallida contains 15 specimens. (TIF) [file pone.0195833.s004.tif]

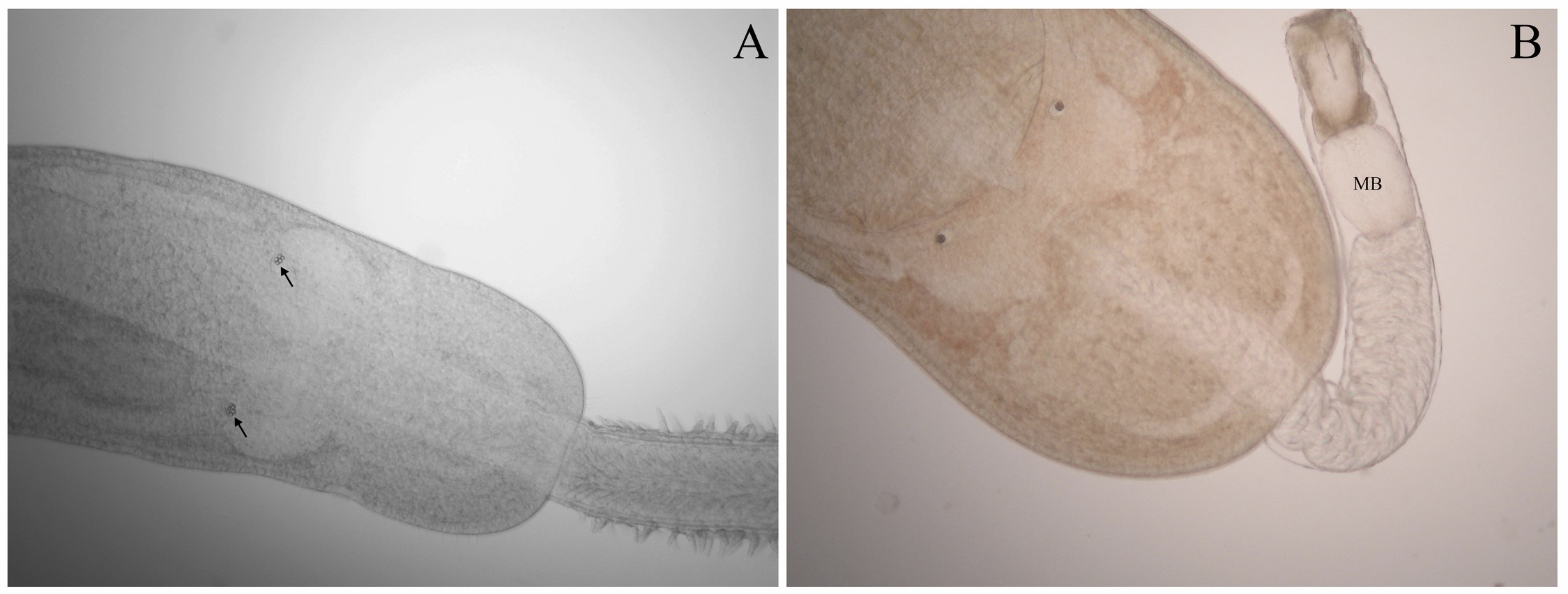

Supplement: S4 Fig — (A) Specimen of Pallida showing statocysts with six granules, marked with arrows. (B) Specimen of Lactea with everted proboscis showing the mid-bulb (MB). (TIF) [file pone.0195833.s005.tif]

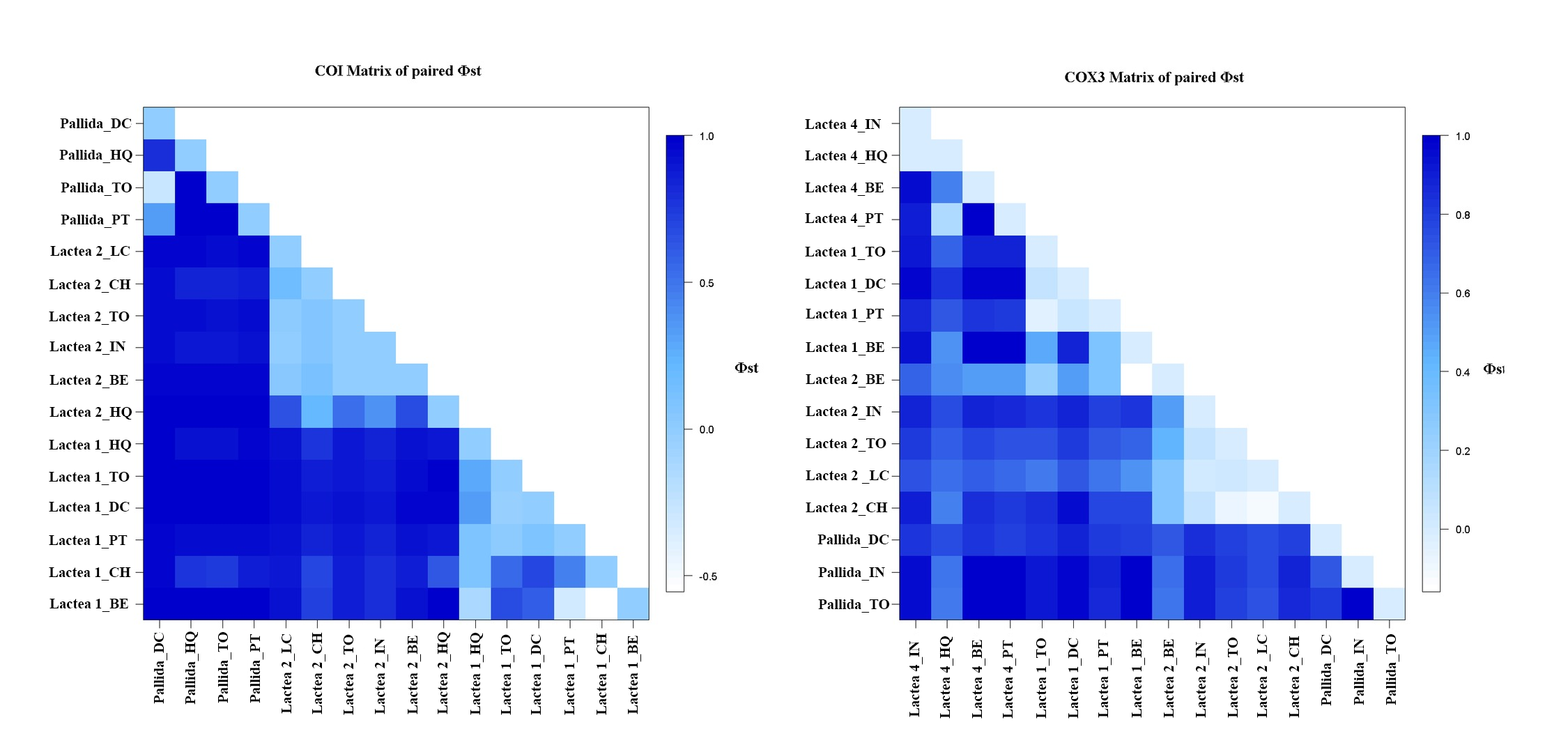

Supplement: S5 Fig — Locality abbreviations as in Fig 1. (TIF) [file pone.0195833.s006.tif]
